# Cardiometabolic risk among rural Native American adults in a large multilevel multicomponent intervention trial

**DOI:** 10.1371/journal.pgph.0001696

**Published:** 2023-07-06

**Authors:** Leslie C. Redmond, Michelle Estradé, Margarita S. Treuth, Caroline R. Wensel, Lisa Poirier, Marla Pardilla, Joel Gittelsohn

**Affiliations:** 1 Dietetics & Nutrition Department, University of Alaska Anchorage, Anchorage, AK, United States of America; 2 Department of International Health, Johns Hopkins Bloomberg School of Public Health, Baltimore, MD, United States of America; 3 School of Health Sciences, Salisbury University, Salisbury, MD, United States of America; University of Louisville School of Public Health and Information Sciences, UNITED STATES

## Abstract

This cross-sectional analysis of the baseline evaluation sample of the Obesity Prevention and Evaluation of InterVention Effectiveness in Native Americans 2 (OPREVENT2) study included 601 Native American adults ages 18–75 living in rural reservation communities in the Midwest and Southwest United States. Participants completed a self-report questionnaire for individual and family history of hypertension, heart disease, diabetes and obestiy. Body mass index (BMI), percent body fat, and blood pressure were measured by trained research staff. About 60% of respondents had a BMI >30 kg/m^2^. Approximately 80% had a waist-to-hip ratio and percent body fat classified as high risk, and nearly 64% had a high-risk blood pressure measurement. Although a large proportion of participants reported a family history of chronic disease and had measurements that indicated elevated risk, relatively few had a self-reported diagnosis of any chronic disease. Future studies should examine potential connections between healthcare access and discordance in self-reported versus measured disease risks and diagnoses.

## Introduction

Lasting colonial impacts on Native American lands, food sources, and traditional means of survival have led to a heavy burden of chronic disease that remains a major driver of health inequity in Native American communities today [1]. The Indian Health Service (IHS) reports that Native American and Alaska Native peoples experience lower life expectancy and disproportionate disease burden, including chronic liver disease and cirrhosis, diabetes mellitus, and chronic lower respiratory diseases [2]. Prevalence of cardiovascular disease (CVD) is also disproportionately higher, and is the leading cause of mortality among Native Americans and Alaska Natives [3]. Cardiometabolic risk factors associated with CVD include hypertension (HTN), type 2 diabetes, hypercholesterolemia, overweight and obesity, and elevated waist circumference and waist-to-hip ratio. In addition, nonalcoholic fatty liver disease (NAFLD), although not assessed here, has now become an important risk factor for several non-communicable diseases, particularly when it is accompanied by obesity and/or diabetes [4]. Many of these risk factors are also disproportionately higher among Native American populations [5]. There is a 10% greater likelihood of HTN diagnosis among Native Americans compared to non-Hispanic whites, adjusting for age [6]. Additionally, 48% of Native American adults are classified as obese based on their body mass index (BMI), compared to 31% in non-Hispanic whites [7]. The causes of these disparities are complex, and may include structural and social determinants as well as inadequate medical and preventive care [2, 8].

Few studies have thoroughly investigated cardiometabolic risk factors among Native Americans [9, 10]. One notable exception is the extensive body of work reporting the findings on obesity, type 2 diabetes, insulin resistance, hypertension, and kidney disease in the Pima Indian population. Nearly half a century of research in this population has led to important understandings of chronic disease development and progression [11] as well as insight into potential cardio-protective characteristics inherent to the population [12, 13]. Despite this wealth of information, there are over 574 federally recognized tribes in the contiguous United States, as well as many without federal recognition and those located in Alaska and Hawai’i, each with their own unique histories, cultures, food patterns, lifestyles, and genetics that contribute to overall health. The relative shortage of data from these tribes in comparison to that obtained from the Pima Indian population highlights the need for more consistent epidemiological monitoring and data reporting on risk factors.

The American Heart Association (AHA) released a scientific statement [14] in May of 2020 concluding that “prevention and treatment of CVD in American Indians and Alaska Natives should focus on control of risk factors and community-based interventions.” To design and implement successful community-based interventions that address CVD, an initial step is to determine prevalence of cardiometabolic risk factors in the target population. The objective of this analysis is to describe the differences between self-reported comorbidities and measured risk factors for cardiovascular disease in rural Native American communities in the Upper Midwest and Southwest US, and to explore regional differences in. Primary outcomes of the analysis included prevalence of self-reported comorbidities and measured risk factors for cardiovascular disease; odds ratios the prevalence of self-reported comorbidities in the Midwest region compared to the Southwest region; and odd ratios for high-risk cardiometabolic measures in the Midwest compared to the Southwest.

## Methods

### Ethics statement

The OPREVENT2 trial received Institutional Review Board (IRB) approval from Johns Hopkins Bloomberg School of Public Health IRB, the Navajo Nation Human Research Review Board and the Indian Health Service (IHS) National IRB, as well as approvals from individual tribal councils. Written informed consent was obtained from all participants.

### Design

This cross-sectional analysis used the baseline evaluation sample from the community intervention trial Obesity Prevention and Evaluation of InterVention Effectiveness in Native Americans 2 (OPREVENT2) [15]. Adults were recruited from six Native American communities in the US: two in the Upper Midwest and four in the Southwest.

### Sample

The OPREVENT2 protocol and intervention have been described in detail elsewhere [15]. The evaluation sample included Native American adults, ages 18–75 years, recruited from household lists provided by each tribal community or through community media from September 2016 –May 2017. Households from the lists were randomly contacted. One adult per household who identified as the main food shopper and/or preparer was invited to participate in the study. Other eligibility criteria included not pregnant or breastfeeding, current tribal member, and planned to stay in the community for at least the next two years. A total of 859 adults were contacted and screened for eligibility; of these, 27% did not meet eligibility criteria (n = 234) and 2.8% refused participation (n = 24). Approximately 100 adults from each of the six different communities participated in the study (n = 601).

All six communities were located in rural areas on Federal Indian Reservation lands. All communities were located in medically underserved areas. The communities in the Southwest had 1–2 food stores per community, one health clinic within a 10-mile radius, and the nearest hospital an average of 73 miles away. The Upper Midwest study communities had 3–4 food stores and 2 health clinics in a 10-mile radius, while the nearest hospital was an average of 20 miles away.

### Measures

#### Self-report

The Adult Impact Questionnaire (AIQ) was a data collection tool that included 144 questions in 12 self-reported sections, including Family and Personal Medical History and Sociodemographics, which were used for this analysis. The AIQ was developed by the research team and designed for use in Native American communities [16]. The Family and Personal Medical History section consisted of two-part questions that first asked if a doctor or nurse had ever told the respondent that they were overweight or obese, had heart disease, high blood pressure/hypertension, or type 2 diabetes. Response options were “Yes,” “No,” or “Don’t Know,” which was treated as “No.” Respondents were then asked if a doctor or nurse had ever told a blood relative that they had any of those conditions.

#### Measured

The AIQ also included a section on six anthropometric measurements performed by trained research staff. Weight (kg), measured using Tanita 300GS (Tanita Corp., Tokyo, Japan) scales, and height (cm) were used to calculate BMI. An estimate for percent body fat (%BF) was obtained using a handheld Omron Fat Loss Monitor (HBF-306). Blood pressure and heart rate were measured using an Omron Automatic Wrist Blood Pressure Monitor, Model #BP652 (HEM-6052-Z). Waist circumference (cm) and hip circumference (cm) were measured according to standard protocol and used to calculate waist-to-hip ratio (WHR). Each measure was taken in duplicate and averaged. A third measure was taken and included in the average in the event that the first two height measurements differed by more than 0.5 inches, weight measurements differed by more than two pounds, and waist and/or hip measurements differed by more than three centimeters. Participants were provided with a health card of their measurements to take home upon completion of data collection. Information included whether each measurement was considered high risk according to established criteria. Research staff were not medical professionals, therefore no medical advice was provided; however, participants were advised to contact their primary care provider if one or more of their measurements was considered to be high risk.

All data collectors participated in a week-long in-person training facilitated by the PI at Johns Hopkins University. Data collectors included tribal or local community members that in the Southwest were fluent in the local tribal language, as well as graduate students from Johns Hopkins University.

### Analysis

Descriptive statistics and analyses were completed using Stata 16SE software (StataCorp LLC, College Station, TX). Pearson’s chi-squared test was used to examine regional differences in categorical sociodemographic factors listed in Table 1, and t-tests were used to examine regional differences in age and BMI. In Tables 2 and 3, logistic regression was used to produce odds ratios comparing the odds of each outcome being reported in the Upper Midwest versus the Southwest (reference population). The regression models controlled for participant age, sex, current smoking status, employment status, and education level. Measured cardiometabolic risk factors were determined to be high risk according to the following criteria: systolic blood pressure >130mmHg or a diastolic blood pressure >80mmHg [17]; waist circumference >102cm for men or >88cm for women [17]; waist-hip ratio of >1.0 for men or >0.8 for women [17]; %BF >36% for men or >38% for women [17]; and BMI >30kg/m^2^ [18].

**Table 1:** Sociodemographic and physical characteristics of OPREVENT2 baseline sample.

Sample Characteristics (n = 601)	Total Sample	Southwest	Upper Midwest
**Sex**			
** Male (%)**	26.3	25.4	32.5
** Female (%)**	73.7	74.6	67.5
**Age (mean years / SD)**	45.8 (15.1)	45.3 (15.5)	46.7 (14.3)
**Education (% with at least GED/High School Diploma)**	82.5	80.8	86.0
**Employed**^**1**^ **(%)**	60.4	55.1*	71.0*
**Current Smoker (%)**	28.7	13.9*	58.0*
**BMI (mean kg/m**^**2**^ **/ SD)**	31.5 (6.3)	31.7 (6.2)	31.2 (6.6)
**Family History of** **≥2 Comorbidities**^**2**^ **(%)**	69.7	61.9*	85.5*

*chi^2^ *p*-value for regional difference < 0.0001

^1^full-time, part-time, seasonal, and self-employed

^2^heart disease, type 2 diabetes, hypertension, and overweight/obesity

OPREVENT2, Obesity Prevention and Evaluation of InterVention Effectiveness in Native Americans 2

**Table 2:** Prevalence of self-reported comorbidities in the OPREVENT2 baseline sample.

Self-Reported Comorbidity	% Total Sample	% in Southwest Communities	% in Upper Midwest Communities	OR (95%CI) for reporting comorbidity in Midwest compared to Southwest^1^
**Overweight/Obese**	49.6	41.4	51.8	1.12 (0.74–1.68)
**Heart Disease**	4.6	4.1	5.5	1.58 (0.60–4.16)
**Hypertension**	31.9	24.7	45.7	3.16 (2.01–4.98)
**Type 2 Diabetes**	19.4	17.3	23.5	1.65 (0.97–2.80)
**≥** **2 Comorbidities**	22.6	19.7	28.5	1.89 (1.14–3.09)

^1^Odds Ratios from logistic regression adjusted for age, sex, education (at least GED/HS diploma), employment (full time, part time, seasonal, or self-employed), and current smoking status

OPREVENT2, Obesity Prevention and Evaluation of InterVention Effectiveness in Native Americans 2

**Table 3:** Prevalence of measured cardiometabolic risk factors in the OPREVENT2 baseline sample.

Cardiometabolic Marker	% Total Sample	% in Southwest Communities	% in Upper Midwest Communities	OR (95%CI) for high-risk measurement in Midwest compared to Southwest^1^
**High-risk BMI** ^**2**^	61.4	60.6	63.0	1.08 (0.71–1.63)
**High-risk Waist Circumference** ^**3**^	81.3	83.4	76.8	0.70 (0.39–1.23)
**High-risk WHR** ^**4**^	79.9	78.8	82.2	3.65 (1.58–8.39)
**High risk % Body Fat** ^**5**^	55.3	58.3	48.5	0.63 (0.38–1.04)
**High-risk Blood Pressure** ^**6**^	63.6	61.3	68.0	1.40 (0.88–2.12)
**≥** **2 High-risk Measures**	87.9	87.8	88.0	1.04 (0.54–2.01)

^1^Odds Ratios from logistic regression adjusted for age, sex, education (at least GED/HS diploma), employment (full time, part time, seasonal, or self-employed), and current smoking status

^2^ > 30kg/m^2^

^3^ > 102cm for males or > 88cm for females

^4^> 1.0 for males or > 0.8 for females

^5^ > 36% for males or > 38% for females

^6^ systolic > 130mmHg or diastolic > 80mmHg

OPREVENT2, Obesity Prevention and Evaluation of InterVention Effectiveness in Native Americans 2

BMI, body mass index

WHR, waist-to-hip ratio

## Results

Table 1 shows characteristics of respondents in the entire OPREVENT2 evaluation sample and by region (n = 601). The majority were female (73.7%). Age ranged from 18 to 75 years, with 48% in the 18–45 year range, and 52% age 46 years or over. Less than 15% had a BMI <25 kg/m^2^ and 10% had a BMI ≥40 kg/m^2^. Approximately 31.6% were not employed and 28.7% were current smokers. Comparing between the Upper Midwest and Southwest regions, about one-third of the sample in the Southwest and Upper Midwest regions were in the obese class I category (BMI >30–34.9 kg/m^2^). A higher percentage of the respondents in the Upper Midwest was employed full-time (67.5%) compared to the Southwest (47.3%). A higher percentage reported being current smokers in the Upper Midwest (58%) than the Southwest (13.9%). Respondents in the Upper Midwest reported significantly higher family history of heart disease, HTN, and type 2 diabetes (*p*<0.01).

Table 2 shows the self-reported comorbidities for all respondents and by region as well as odds ratios for reporting each comorbidity in the Midwest compared to the Southwest. For all respondents, self-reported comorbidities ranged from ~5% for heart disease to ~50% for overweight/obesity. Nearly 23% of respondents reported two or more comorbidities, and the odds of reporting at least two comorbidities was significantly greater in the Midwest compared to the Southwest.

Table 3 shows the prevalence of measured cardiometabolic risk factors as well as odd ratios for having a high risk measurement in the Midwest compared to the Southwest. Over half of the study population had a BMI or %BF classified as high risk, and approximately 63.6% had a blood pressure measurement classified as high risk. Approximately 80% had a waist circumference and waist-to-hip ratio classified as high risk. A majority of the sample (87.9%) had two or more measurements classified as high-risk.

## Discussion

### Summary

Self-reported measures are frequently used to determine CVD prevalence and population health services needs within communities [19]. Our analysis revealed a discordance between the prevalence of self-reported comorbidities and measured risk factors for CVD. Around one third of respondents reported having no comorbidities; however, measurements of BMI, WHR, and %BF obtained by research staff classified nearly 60% of the study population as having obesity, nearly 80% as high risk for WHR, 64% as high risk for blood pressure, and 55% as high risk according to %BF. This magnitude of observed discordance within this population has not been previously documented in the literature to our knowledge. Compounding this discordance was the remarkably higher prevalence of self-reported comorbidities in the Upper Midwest communities compared to the Southwest communities.

Prevalence of self-reported comorbidities ranged from ~5–50% while the prevalence of a positive family history of comorbidities was much higher, with nearly 70% of the sample reporting a family history of two or more comorbidities.

The anthropometric and blood pressure measures taken in the communities revealed that participants were at high risk for HTN and other cardiometabolic risk factors. The proportion of those classified as high risk ranged from ~55% (for %BF) to 80% (for high waist circumference). Variations in prevalence of high-risk measures existed between the two regions, but in general the trend was the same: that about half of the participants were at an increased risk of several adverse health outcomes, including obesity, HTN, and type 2 diabetes. Additionally, approximately 77% of the sample was classified as high risk in three or more measurements. By comparison, a report of measured risk factors for a southern Native American tribe found that only 21.3% had three or more risk factors [20].

The number of measured high risk cardiometabolic markers in the OPREVENT2 communities was consistent with the high prevalence of HTN and diabetes in Native American populations [3]. However, there was a notable difference between the measured high risk cardiometabolic markers and the self-reported comorbidities. This observation is concerning, especially given the importance of cardiometabolic factors and their contribution to increased disease risk, incidence, and overall burden. Management of these risk factors, which are generally considered to be modifiable either through lifestyle changes, pharmacotherapy, or a combination thereof, offer one of the most attainable ways to prevent and treat CVD. Early awareness of these risk factors, screening, and diagnosis have been identified as being fundamental to reducing, delaying, and preventing cardiometabolic risk [21]. For example, losing just 5% to 10% of body weight may reduce risk of CVD in high risk populations [22] and blood pressure reduction has consistently been shown to significantly reduce risk for CVD and associated outcomes [23]. The low self-reporting of comorbidities in the study population, combined with the high prevalence of measured high risk cardiometabolic factors, indicates a missed opportunity for early detection, management, and treatment, possibly resulting in worse health outcomes over time that could have been prevented. The observed discordance may be indicative of several underlying issues, including access to and availability of culturally appropriate preventative services, diagnostic care, and treatment options as well as cultural competence among providers, variation in health literacy among patients, and miscommunication leading to misunderstandings between patients and providers.

Access to health care can be a major concern among Native American populations. Many Native American reservations are located in rural areas with limited access to resources like grocery stores [24] and health clinics [25]. Several studies show that reservation-dwelling Native American adults report cost and insurance barriers, distance to medical facilities or specialists, long wait times, and transportation difficulties as barriers to care [26]. A 2019 integrative review of barriers to health care access among American Indians and Alaska Natives in rural America reported that rural residence or geographic isolation limited individuals’ abilities to access quality health care, resulting in poor health outcomes [27]. Reservations are also frequently designated as Health Professional Shortage Areas (HPSA) [28], and Native Americans have been designated as a Medically Underserved Population (MUP) by the Health Resources and Services Administration (HRSA) Bureau of Health Workforce [29]. According to the HRSA, MUPs face a shortage of primary care health services and may also face economic, cultural, or language barriers to health care [30]. Although there are several existing efforts to improve medical services on rural reservations, such as the IHS Scholarship Program and the Loan Repayment Program, the average medical officer vacancy rate across all IHS service areas remains high at 25% [30]. These barriers may contribute to chronic disease in these populations [31, 32], including the OPREVENT2 sample. It is possible that these barriers had prevented respondents from seeking health care for extended periods of time and that risk factors had been allowed to appear and progress unnoticed. This might explain why some respondents self-reported no risk despite documentation of measured risk by OPREVENT2 staff. Additionally, while all OPREVENT2 communities were rural and considered medically underserved populations, it is possible that the Upper Midwest communities had other barriers to accessing preventive health care and/or treatment, which may help to explain the significantly greater prevalence of self-reported comorbidities in those communities. In the entire sample, this suggests that there is a need to support increasing prevention efforts and diagnosis in these rural communities.

The discordance between measured high risk cardiometabolic markers and self-reported comorbidities may also be the result of poor communication and understanding of disease risk and diagnosis between providers and patients, possibly related to health literacy, or the ability of individuals to obtain, process, and understand basic health information and services needed to make appropriate health decisions [33]. Increasing health literacy is critical and has been a goal of tribal organizations funded by the Centers for Disease Control as part of the Good Health and Wellness in Indian Country from 2014–2019 [34]. Health literacy is complex, and may interfere with a patient’s ability to seek medical care at multiple levels of healthcare delivery [35]. Oftentimes attempts to communicate health information are complex, use formats that are difficult to understand, try to communicate too much at once, and may ultimately distort the message [36]. Patients who have difficulty comprehending health instructions may avoid seeking health care altogether [33]. The AHA has identified the role of health literacy in primary and secondary prevention of CVD [35], including: the recognition and knowledge of HTN; knowledge, self-efficacy, self-care, and attitudes and beliefs related to type 2 diabetes (including a strong relationship between health literacy and diabetes mellitus knowledge in American Indians and Alaska Natives) [37]; knowledge and self-efficacy related to obesity, dietary choices, and exercise; tobacco use and cessation; poorer outcomes preceding and following coronary events and decreased adherence to medications preceding admissions; and higher all-cause mortality and a barrier to successful self-care among patients with heart failure [35]. A recent systematic review determined that there are significant differences in health literacy between rural and urban populations, with lower health literacy in rural groups [38]. Of the 19 articles reviewed, just over half concluded that rurality itself was not a significant determinant, but rather confounding variables including: education, age, gender, socioeconomic status and race/ethnicity [38]. Regardless of whether living in rural areas is the reason for disparities in health literacy, it is important for providers and communities to recognize that the disparity exists and to work towards solutions that address the underlying causes.

Poor cultural competency of providers may also contribute to poor health outcomes or hesitance to seek health care services among members of culturally diverse communities. Cultural competence emphasizes patient-centered care that is focused on respect for the patient and differences in health-related values and beliefs, shared decision making, and strong patient-provider relationships [39]. Lack of cultural competence may lead some individuals to experience racial discrimination and cultural misunderstandings during health care encounters [26]. For example, Native American women from one Gulf Coast tribe reported rushed or rude provider interactions, providers not listening or ignoring their concerns, inadequate care or diagnosis, and need for personal relationships with providers [40]. Minimal representation within the medical profession may also contribute to issues with cultural competency. The number of Native American physicians is extremely low at only 0.4% of all physicians and has been found to be declining [41]. Finally, there is a shortage of culturally appropriate health programs and messages. Native American populations have historically been excluded from many national public health nutrition programming initiatives; for example, federal food and nutrition assistance programs provide little access to traditional foods and limited opportunities to remain connected to traditional foodways [42]. Recognizing and addressing these shortcomings relies on improved cultural competency of health care providers and public health professionals.

Although we did not specifically measure the impact of rurality, the health literacy of respondents, or the cultural competency of providers in the OPREVENT2 communities, it is reasonable to hypothesize that these issues may have played a role in the discordance between self-reported comorbidities and measured risk factors that was observed. Strengthening the patient-provider relationship and building a shared understanding of risk, diagnosis, and treatment, may help minimize miscommunications and misunderstandings. It is important for medical practitioners in these services areas to prioritize availability of culturally-tailored health information and include Native American patients in medical discussions and decisions [36].

### Limitations

The original primary aims of the OPREVENT2 trial did not include examining differences in self-reported comorbidities and measured high risk metabolic markers, and the data collection instruments were therefore not designed to make direct comparisons between self-reported comorbidities and measured risk factors. There was also no survey question to assess how many respondents had been unaware of their high-risk metabolic markers after receiving their measurements. This information could have added depth to this analysis and insight into causes of the observed discordance between self-reported comorbidities and measured risk factors, and collecting these data should be considered for future research in this population.

Although the AIQ had been successfully implemented in similar populations in previous trials, there was anecdotal evidence from data collectors that some respondents may have misunderstood some of the questions about personal and family medical history. For example, there may have been confusion about the types of diabetes (type 1, type 2, and gestational diabetes) or differing interpretations of “blood relative,” as some Native American communities consider members of the same tribe or clan to be blood relatives. Additionally, this measure may be limited because it does not consider the “closeness” of a relative, for example a parent/sibling versus a cousin, or number of blood relatives with the condition. Some respondents may have underreported a “heart disease” diagnosis if their physician had used different, more specific terminology, such as coronary artery disease or atherosclerosis. In addition, among those reporting a diagnosis, the survey did not allow for determination of treatment efficacy.

Finally, the data presented here may not be generalizable to other Native American populations, which vary widely in culture, genetics, and dietary practices.

## Conclusion

In conclusion, this study demonstrated a discordance between self-reported comorbidities and measured high risk cardiometabolic markers. As well, there was a notably high prevalence of comorbidities reported in the family histories. Future studies should explore the impacts of access to care, health literacy, and cultural competence on the reporting and diagnosis of cardiometabolic risk factors in these Native American MUP communities. Implications for practice include prioritizing efforts among healthcare providers to improve cultural competency and build stronger patient-provider relationships to overcome barriers due to health literacy and minimize miscommunications. Healthcare organizations in these communities should also focus on recruitment and retention of skilled medical officers to decrease the medical officer vacancy rate. Future policies should aim to increase funding allocated to HPSAs and MUPs and support opportunities for culturally appropriate chronic disease prevention program and intervention development. Finally, tribal leaders may consider a shift towards a healthcare delivery system that is under the control and direction of local Native American communities that will provide them with more control and decision-making authority and the flexibility to prioritize programs to meet the most salient health needs of their communities [25].
